# Gleanings from Our Exchanges

**Published:** 1873-01-15

**Authors:** 


### Gleanings from Our Exchanges.

### DIFFICULTY IN DIAGNOSIS OF TUBERCULAR MENINGITIS.

Dr. Peter (*Jour. de Med. et Chir. Prat.*) says that meningitis is a pregnant source of error in diagnosis, and we ought to be on our guard against such. Thus, a patient entered hospital under violent delirium, which had burst forth without any warning, and the diagnosis was made *delirium tremens.* Yet the autopsy showed that the patient had tubercular meningitis. Generally, tubercular meningitis commences with pain, convulsion, strabismus, etc., all its symptoms in a word, whilst the intelligence is intact. Here the contrary had taken place, because the patient was a confirmed drunkard, and that, when the cerebral lesion came on, it had changed his disease into delirium tremens. In these difficult cases for diagnosis, it is important often to look to one symptom, to the antecedents, and to study closely the whole of the phenomena; if in doubtful cases we arrive at finding a meningeal ailment, we have a great presumption gained.

Sometimes the commencement of the disease is marked solely by repeated vomiting, without elevation of pulse, or any other symptom. We then inevitably think of indigestion, and the error is only dispelled by the progress of events. A child, æt. 18 months, was attacked with retention of urine, and remained forty-eight hours without micturating. This was not a case of anuria of infants with fever, for the bladder became distended, and was emptied by the sound. There was true constipation of the bladder present. M. Peter, called in in twenty-four hours, found that the child — offspring of a delicate mother — was crying in a peculiar way, whilst its face was covered with a blush. The "cerebral spot" could be made to appear, although ill-marked, and there was a very feeble convulsive shock. He made the diagnosis of tubercular meningitis, which was soon justified by the termination of the disease.

On another occasion he was called to the neighborhood of Paris, for a child convalescent from measles. The left side of the body had one day commenced to be palsied, without signs of apoplexy, and in twenty-four hours the palsy was progressively completed. However unaccustomed the commencement appeared, in the absence of all possible supposition, and in view of the antecedents, finding the child delicate, M. Peter thought of meningitis. The abdomen was retracted, and he noticed a well-marked mark of meningitis. He decided for a tubercular meningitis, and attributed the hemiplegia to accumulation of tubercular granulations at one point of the base of the brain having caused a small ficus of softening. In fact, soon the collection of symptoms developed themselves, and the child died with all the signs of cerebral meningitis.

Although constipation is an almost constant symptom, it may be wanting. M. Peter was called to a delicate child, æt. 3 years, who had been somnolent for some days, and attacked with abundant diarrhœa. The state of coma was so profound that when the child was shaken it did not move. M. Peter diagnosed tubercular meningitis, in spite of the diarrhœa. We may consider the disease for some days as tubercular peritonitis. The child fell away rapidly, its cries became more characteristic, and the meningeal spot could be noticed clearly with cutaneous hyperæsthesia. One day convulsions with irregular pulse supervened, and death with epileptiform attack.

This short collection of unusual cases shows the necessity of grouping any symptoms which are noticed. In this case, the meningeal stain, which is provoked by scratching with the nail the skin of the abdomen, chest, or limbs, and which persists pretty long after the scratch, becomes a symptom of importance. Taken by itself, its value is infinitely lessened.—*The Doctor.*

Relation of Bad Smells to Disease.—Some months ago an item appeared in this journal (*Pacific Medical and Surgical Journal*), headed "Needless panic about sewers," presenting a remarkable instance of the innocuousness of sewer effluvia. Our New York correspondent—the one who hates tobacco—takes up the matter with his usual vim, as follows:

"Needless panic about sewers" is too positive for an obscure case. Open to argument, agreed. But you *do n't know enough* to justify a dogmatic statement. Those fellows who worked in the sewers, did n't *sleep* in 'em. Men at work are always perspiring—*throwing off*, and therefore impervious to attack, in a general way. Arsenic diggers live long in mines, and eat arsenic. That do n't prove that arsenic and its fumes won't kill people—does it? In some states of the atmosphere, even the little waste holes in our chamber basins, let up such a horrid stench that the room is intolerable. I have frequently cured, or stopped it, by pasting paper over them. Are not people made sick with the smoke of tobacco? What *is* stench? Who knows? Do n't sewers give out a malarious vapor? In southern Indiana people do n't go out after sundown in summer, because ague follows *sure*. What's that? I would rather risk advocacy of the poisonous or deleterious character of all foul odors *a priori*, than the other side. Science and purity must accord here, I think. Some people get sick with the odor of apples. The "rose fever" or "hay fever" is quite common—comes on with some on the 28th day of July, and never fails on *that day*. Dr. Mason, scientific man near me, has the hay fever now, which, however bad, disappears as soon as he gets on the water in a ferry-boat, and returns when he touches the land. I have no doubt that there is a specific poison to many lungs in the exhalations of our sewers—quite independent of the mere displacement of wholesome air. Anyhow, it strikes me that the *Journal* commits itself in an unscientific manner. Some big landlord of a sewer district has paid a doctor for a special evidence, which, to my mind, proves nothing. I believe it is the theory of all our best physicians here, that all putrid exhalations are unwholesome *in*halations, and therefore in degree poisonous. The same argument suggested by the *Journal* would prove tobacco and rum innocuous—or, at least, it squints so, or it seems to squint so, or it seems to seem to squint that way.

Atropia in Night-Sweats. — Dr. J. C. Wilson, in the Philadelphia *Medical Times*, calls attention to the efficacy of atropia in arresting the night-sweats of phthisis, in doses of one-sixtieth of a grain once or twice a day. It was promptly successful after the failure of sulphuric acid, tannic acid, oxyd of zinc, and other remedies. Dr. Sidney Ringer also furnishes similar testimony in the *Practitioner*, he having injected it in the skin in doses of the hundredth part of a grain. Dryness of the fauces and dilatation of the pupils result from a continuance of the treatment.—*Pacific Med. Jour.*

Population of the Globe.—There are on the globe 1,288,000,000 souls, of which 360,000,000 are Caucasians; 522,000,000 are Mongolians; 190,000,000 are Ethiopians; 176,000,000 are Malays; 1,000,000 are Indo-Americans. There are 8,642 languages spoken, and 1,000 religions. The yearly mortality of the globe is 42,043,000 persons. This is at the rate of 115,200 per day, 4,800 per hour, 80 per minute. Among 10,000 persons, one arrives at the age of 100; one in 500 attains the age of 80; one in 100 to the age of 70. In 100 persons, 95 marry.—*Medical Record.*

Medical Instruction for Women in Russia.—Next January a special course of instruction for midwives will be commenced at the Imperial Academy of Medicine and Surgery at St. Petersburg. Applicants must not be under twenty, and, if still under the authority of parents, must present the written consent of the latter. They will be submitted to a preliminary examination, especially with reference to their knowledge of mathematics, within the limits of instruction in the gymnasium for young girls, their intellectual development, and their knowledge of Russian. The object of the course is to prepare trained midwives to give necessary medical assistance to parturient women, and to treat diseases of women and children. The course will last four years, and the fees will be fifty roubles per annum.

Amaurosis Cured by Electricity.—Dr. Ramorino (*La Nuova Lig. Med.* No. 26) says that he has cured in his eye dispensary in Genoa, with three applications of the electric current, a youth of 25, a mason, affected with anæsthesia of the retina of the right eye, after a blow from the point of an umbrella.

A New Scotch Lunatic Asylum.—Glasgow, Barony Parish, Scotland, is to have a new lunatic asylum, costing about $300,000. The grounds comprise 176 acres, of which some twelve acres will be covered with buildings.

Electricity as a Means of Resuscitation.—Allan McLane Hamilton, M. D., New York (*Am. Practitioner*, Oct., 1872), in a paper on this subject, speaks of the following results arrived at from his own practice and experiments: 1st. That it is useless to expect good results if five minutes have elapsed since life appears extinct. 2d. That the current should be applied faithfully and steadily, one pole being placed on the ensiform cartilage, the other on the base of the skull or over the tracks of the great nerves of the neck. 3d. That the faradic and interrupted galvanic currents are the best. 4th. That the current should be applied some time after respiratory movements have become regular.

In concluding, the author says: The necessity of having a battery within reach is apparent. Every practitioner should have a small one for emergencies. They should be kept at each life-saving station on the coast, ready charged, with directions for immediate use. If this were done, he doubts if the percentage of deaths would be so great as it now is. Artificial respiration by the production of muscular movements is a very valuable means of restoration; but a force that acts directly upon the nerves supplying the muscles of respiration, is by far the surest and best.

Anæsthetics.—According to John Morgan, M.D., F.R.C.S.I., Surgeon to Mercer's Hospital, Dublin (*British Med. Journal*, October, 1872), the ratio of deaths following the employment of anæsthetics is as follows:

| Agents Employed. | Deaths. | Inhalations. |
|---|---|---|
| Ether, - - | 4 to 92,815, or 1 in | 23,204 |
| Chloroform, - | 53 to 152,260, or 1 in | 2,873 |
| Mixture of Chloroform and Ether, | 2 to 11,176, or 1 in | 5,588 |
| Bichloride of Methylene, - | 2 to 10,000, or 1 in | 5,000 |

Therapeutical Applications of Eucalyptus Globulus.—At the last meeting of the San Francisco Medical Society, Dr. Pignedupuytren said he had used the eucalyptus in the French Hospital for a year, during which time many interesting results had been noted. In March last, a hundred of the small trees had been planted on the Hospital grounds. They had now reached the height of seven feet. He related the following cases:

A man had arteritis of the leg, succeeded by gangrene, which extended so high up as to render amputation impossible. In two weeks a large ulcer resulted, whose odor was horribly fetid. Everything in turn was employed to destroy this odor, to no effect. At last a decoction of eucalyptus was resorted to, and, without exaggeration, in five minutes all fetor had disappeared. The decoction continued to be used with the same effect until death occurred, two or three weeks subsequently.

Another man, who had been under treatment in the Hospital for two months, with extensive, deep ulcer from varix, of a year's duration, had the decoction applied to the ulcer three times a day, with remarkable effect. In five or six days the ulcer was entirely covered with healthy granulations, and in a month it was entirely well.

A woman had been troubled for many months with an ulcer around the orifice of the urethra. It was cauterized five times with no result. After twelve days' use of the decoction of eucalyptus, washing thrice daily, it was well.

Four cases of syphilitic chancres healed under the eucalyptus dressing in five or six days, without other treatment. These were very recent cases, or constitutional treatment would have been resorted to.

He had but one case of intermittent fever to report. This had proved rebellious to quinia, and also to arsenic, which latter had been administered for two weeks. A three weeks' course of the eucalyptus cured entirely.

So numerous were the cases of bronchitis cured with the drug, that it was hardly worth while to mention them. A syrup made by adding sugar to the decoction, and evaporating, was here preferred.

The decoction was made by boiling twelve large leaves (green) in a pint of water down to one-third. The tincture was prepared from the dry leaves.

Dr. Pigne-dupuytren added that he now always prescribed quinia in the infusion of eucalyptus, and considered such combination of great efficacy.—*Pacific Medical Journal.*

The Ether and Chloroform Controversy.—Within the last year the number of deaths from chloroform has increased so alarmingly as to excite a more extensive hostility to its use than has ever before existed. The movement is simultaneous in Great Britain, on the Continent, and in America. M. Diday, of Lyons, in addressing the editors of the *Gazette Hebdomadaire*, refers to three deaths reported in a single week by the London *Medical Press*, and contrasts this experience with that of Lyons, where ether was substituted for chloroform many years ago, and where the profession are well satisfied with the change, and their patients still more so.—*Pacific Med. Jour.*

